# Characterization of biofilm matrix, degradation by DNase treatment and evidence of capsule downregulation in *Streptococcus pneumoniae *clinical isolates

**DOI:** 10.1186/1471-2180-8-173

**Published:** 2008-10-08

**Authors:** Luanne Hall-Stoodley, Laura Nistico, Karthik Sambanthamoorthy, Bethany Dice, Duc Nguyen, William J Mershon, Candice Johnson, Fen Ze Hu, Paul Stoodley, Garth D Ehrlich, J Christopher Post

**Affiliations:** 1 Center for Genomic Sciences, Allegheny-Singer Research Institute, Pittsburgh, PA 15212, USA; 2 Department of Microbiology and Immunology, Drexel University College of Medicine, Allegheny Campus, Pittsburgh, PA 15212, USA; 3 Tescan USA Inc, 508 Thomson Park Drive, Cranberry Township, PA 16066, USA

## Abstract

**Background:**

*Streptococcus pneumoniae *is a common respiratory pathogen and a major causative agent of respiratory infections, including otitis media (OM). Pneumococcal biofilms have been demonstrated on biopsies of the middle ear mucosa in children receiving tympanostomy tubes, supporting the hypothesis that chronic OM may involve biofilm development by pathogenic bacteria as part of the infectious process. To better understand pneumococcal biofilm formation six low-passage encapsulated nasopharyngeal isolates of *S. pneumoniae *were assessed over a six-eight day period *in vitro*.

**Results:**

Multiparametric analysis divided the strains into two groups. Those with a high biofilm forming index (BFI) were structurally complex, exhibited greater lectin colocalization and were more resistant to azithromycin. Those with a low BFI developed less extensive biofilms and were more susceptible to azithromycin. dsDNA was present in the *S. pneumoniae *biofilm matrix in all strains and treatment with DNase I significantly reduced biofilm biomass. Since capsule expression has been hypothesized to be associated with decreased biofilm development, we also examined expression of *cpsA*, the first gene in the pneumococcal capsule operon. Interestingly, *cpsA *was downregulated in biofilms in both high and low BFI strains.

**Conclusion:**

All pneumococcal strains developed biofilms that exhibited extracellular dsDNA in the biofilm matrix, however strains with a high BFI correlated with greater carbohydrate-associated structural complexity and antibiotic resistance. Furthermore, all strains of *S. pneumoniae *showed downregulation of the *cpsA *gene during biofilm growth compared to planktonic culture, regardless of BFI ranking, suggesting downregulation of capsule expression occurs generally during adherent growth.

## Background

*Streptococcus pneumoniae *is an important bacterial pathogen worldwide that causes localized disease including pneumonia and otitis media (OM), as well as invasive infections such as septicemia and meningitis. The ability of this organism to persist in the respiratory tract and transition between asymptomatic carriage and infection stimulates intense research interest in *S. pneumoniae*. Pneumococcus is a leading bacterial cause of acute OM in children where it is estimated that by age five, over 80% of children have had at least one OM episode [1]. *S. pneumoniae *is also frequently detected in chronic otitis media with effusion (OME) [2], the most common cause of acquired conductive hearing loss in children. While, invasive disease has decreased with the introduction of the pneumococcal heptavalent conjugate vaccine (PCV7), localized infection in the middle ear has not been reduced as dramatically and new serotypes, some resistant to multiple antibiotics, have emerged [3,4].

The detection of pneumococcal-specific DNA and RNA in culture-negative effusions in multiple studies suggests that active bacterial infections are present more frequently than culture results indicate [2,5-7], and both the persistence of bacteria and recalcitrance to antibiotic treatment in OME suggest that chronic OM may be associated with bacterial biofilm development on the mucosal surface of the middle ear [7,8]. This hypothesis was recently supported by evidence of adherent *S. pneumoniae*, *Haemophilus influenzae *and *Moraxella catarrhalis *directly on the middle-ear mucosal epithelium (MEM) in children receiving tympanostomy tube (TT) placement for chronic otitis media [9]. In this study, clusters of adherent pneumococcus were observed on the MEM using 16S rRNA fluorescent *in situ *hybridization (FISH) and anti-pneumococcal immunostaining. Pathogenic biofilm bacteria were absent on MEM biopsies from patients undergoing surgery for cochlear implantation, suggesting that adherent bacteria are not typically present on MEM.

Biofilm development is initiated when bacterial cells attach to a surface, proliferate and extrude a complex extracellular matrix that binds cells together and to a surface. Several chronic infections, such as cystic fibrosis pneumonia, and chronic tonsillitis and sinusitis, exhibit biofilm development in the respiratory tract of the human host [10-14]. Bacteria within biofilms exhibit two fundamental characteristics: production of an extracellular polymeric substance (EPS) matrix and increased resistance to antimicrobial treatment [11,12]. Depending on the type of bacteria in the biofilm, the EPS can be made up of polysaccharides, proteins and DNA [15]. Following attachment and biofilm development, bacteria may undergo significant phenotypic shifts including induction of different metabolic pathways, reduced cell division, and development of resistance to antibiotic concentrations capable of killing planktonic bacteria [16-19]. Biofilm formation is important in understanding the extent of bacterial phenotypic plasticity in response to varying environmental conditions and several papers have reported pneumococcal biofilm formation *in vitro *under various growth conditions [16,20-26].

The pneumococcal capsule is considered a major virulence factor. Capsule expression is thought to interfere with biofilm formation [20,23,24,26] and biofilm development may select for unencapsulated phenotypic variants [20,23,26]. However, *S. pneumoniae *is also known to phenotypically vary capsule production upon adherence to epithelial cells [27]. The polysaccharide capsule-specific regions of *S. pneumoniae *are encoded by a cluster of genes located between *dexB *upstream and *aliA *downstream [28] and the capsule is predicted to be transcribed as a single operon, initiating upstream of the most conserved gene in the operon, *cpsA *[29].

We chose a static culture system to examine biofilm formation under conditions that simulate those in the middle ear during OME. Biofilm development in six encapsulated clinical strains of *S. pneumoniae *representing six different serotypes was assessed by three independent techniques and statistically analyzed to formulate a parametric index of biofilm development that resulted in ranking the strains into two groups. The index was then used to test hypotheses concerning pneumococcal biofilms such as the composition of the extracellular matrix, susceptibility of pneumococcal biofilms to antimicrobial treatment and capsule expression using *cpsA*.

## Results

### *S. pneumoniae *clinical strains vary in initial attachment, kinetics of biofilm formation and biofilm structural complexity and the Biofilm Forming Index (BFI)

**Figure 1:**
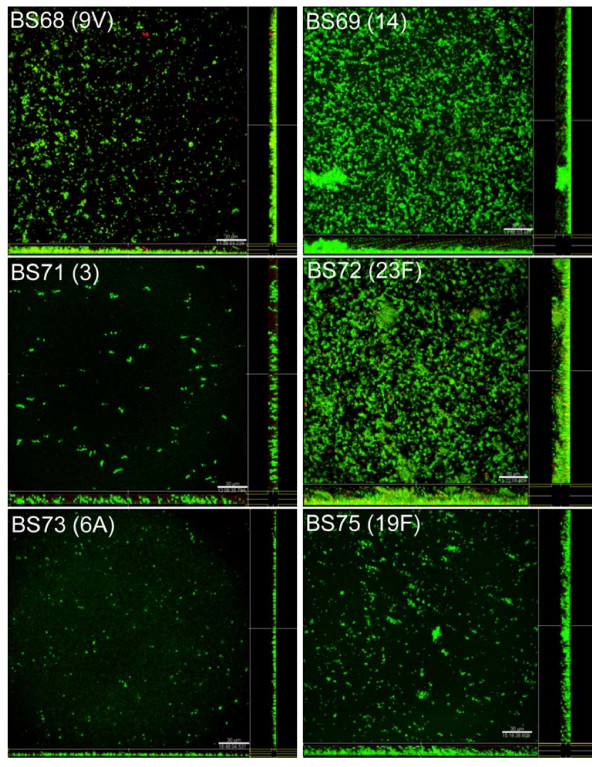
**CLSM images of biofilm development by clinical isolates of *S. pneumoniae *stained with *Bac*Light after 6 days of culture showing viable (green fluorescence) and nonviable (red fluorescence) pneumococci within the biofilms.** Images are maximum projections or reconstructed confocal stacks consisting of a series of x-y sections. Sideviews (YZ – left and XZ – bottom) are saggital sections of the biofilm. Scale bar = 30 μm.

Pneumococcal biofilm development was examined *in situ *over time for each strain using the BacLight Kit and CLSM. All isolates developed biofilms over the 6 day period. However, there was considerable variability in both the extent and kinetics of biofilm development by the isolates, and in the number of viable and nonviable cells (Fig. 1). After 24 hours all 6 pneumococcal isolates had formed heterogeneous biofilms consisting of individual cells, small chains and small clusters of lancet-shaped cells with strain BS72 exhibiting the greatest number of attached cells and a complex biofilm architecture at this time point consisting of small clusters and towers (data not shown). By day 3, strains exhibited a range of ultrastructural characteristics from individual cells and small microcolonies stippled across the surface (BS68, BS71 and BS73) to clusters of bacteria in large towers attached to the substratum (BS69, BS72 and BS75) (data not shown). Strains BS69, BS72 and BS75 developed the most extensive biofilm architecture by day 6 of culture, growing in tall towers of viable cells up to 25 μm in height over the surface (Fig. 1). Time lapse CLSM imaging through the thickness of biofilm towers in real time showed that the towers were free to oscillate indicating that pneumococcal biofilms were dynamic in the fluid (see movie at ). In contrast, strains BS68, BS71 and BS73 exhibited smaller cell clusters (5–10 μm), fewer towers and less extensive surface coverage and ultrastructure. Nevertheless, biofilms formed by these strains exhibited numerous microcolonies of viable cells attached on the surface.

**Figure 2:**
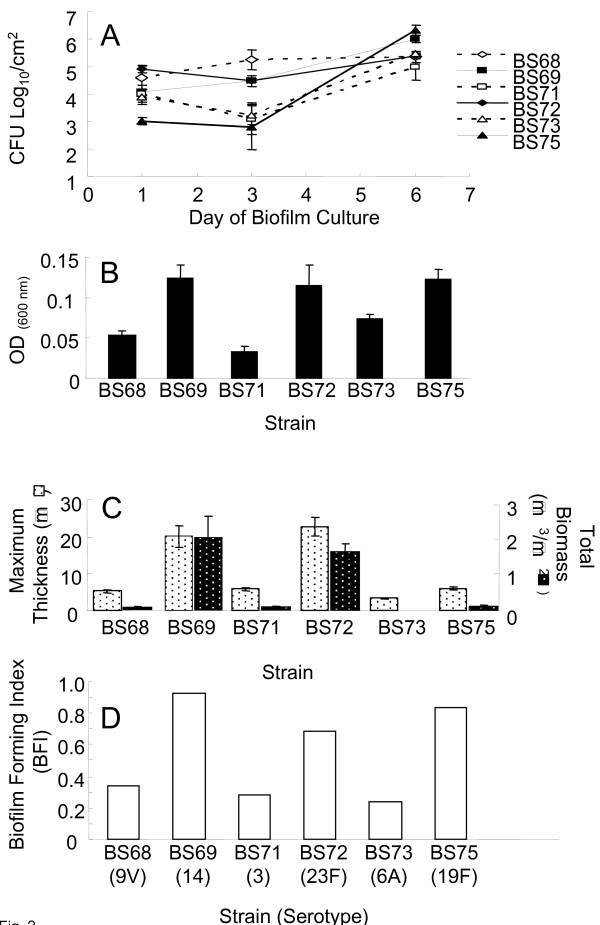
**Quantitative assessment of biofilm development by clinical pneumococcal isolates. Fig. 2A. **Biofilm development by clinical isolates at days 1, 3 and 6 of culture on polystyrene plates as shown by viable adherent cells (CFUs/cm^2^). Points represent an average of three duplicate wells per time point in two independent experiments. Error bars represent SD. **Fig. 2B. **Biofilm development (initial attachment) assayed by crystal violet absorbance comparing 6 clinical pneumococcal isolates on polystyrene over 24 hours. Bars show average triplicate samples of 5 independent experiments. Bars represent SD. **Fig. 2C. **COMSTAT assessment of pneumococcal biofilm development after 6 days of culture. Two parameters of surface attached pneumococci are shown: maximum thickness (biofilm towers) (left axis) and biomass (biofilm volume) (right axis). Bars represent an average of 3–5 images taken from duplicate plates in 2 independent experiments (minimum n = 12). Error bars represent standard error of the mean. Fig. 2D. Biofilm forming index (BFI) of the 6 pneumococcal clinical strains (serotype in parentheses) combining statistical analyses from the widely used biofilm assays: CFU/cm^2^, CV assay and COMSTAT analysis. The index ranks each strain according to biofilm formation.

To better assess biofilm development quantitatively, CFUs of attached pneumococci were enumerated at 3 time points to assess the kinetics of biofilm development. Viable adherent pneumococci increased over time in all strains. Attached viable pneumococci were present for all clinical isolates at day 1 ranging from 3.1_(log10) _CFUs/cm^2 ^for BS75 up to 4.9_(log10) _for BS72 (Fig. 2A). BS75 biofilms contained significantly fewer culturable cells and BS72 biofilms contained a significantly greater number of cells than all of the other 5 strains at day 1, confirming CLSM observation. By day 6, there was a significant difference in the number of adherent cells among the clinical isolates; BS69 and BS75 showed the most viable attached pneumococci with an average CFU/cm^2 ^of 6 and 5.7, respectively. BS72 averaged 5.4 log_10 _CFUs/cm^2^, however the number of CFUs/cm^2 ^for this strain was not statistically different from BS68, BS71 or BS73 biofilms, which demonstrated average viable attached pneumococcus on the order of 5.4, 5.0 and 5.5 log_10 _CFUs/cm^2^, respectively. Although the kinetics of surface attachment varied among clinical pneumococcal isolates, viable adherent cells increased over 6 days in all strains.

All 6 clinical isolates also attached over a 24 hour period (Fig. 2B) demonstrated by crystal violet staining. However, strains BS69, BS72 and BS75 produced nearly twice as much biofilm as strains BS68, BS71 and BS73. BS71 formed significantly less biomass than all of the other isolates in this assay (P values < 0.05). There was no significant difference between BS72, BS69 and BS75.

Strain differences were also quantified using COMSTAT (Fig. 2C). Biofilm biovolume (biomass) and maximum thickness were lower for the clinical strains BS68, BS71 and BS73. Consistent with the experimental results of the CFU/cm^2 ^and CV assays, BS69 and BS72 exhibited the greatest extent of total biomass and maximum thickness (> 20 μm). Surface roughness, a measure of biofilm heterogeneity, was low for these isolates, with greater heterogeneity correlating with microcolonies separated by large voids (data not shown). ANOVA analysis of COMSTAT data demonstrated significant differences among the strains with respect to biofilm formation. Comparison of averages from grouped COMSTAT data (multiple plates, multiple experiments) demonstrated that BS69 and BS72 exhibited significantly more biomass than the other strains in this biofilm assay.

When data were combined from all 3 biofilm assays using the combined biofilm forming index (BFI) metric, the strains divided into two groups (Fig 2D). Strains BS69, BS72 and BS75 demonstrated far greater biofilm development (in order of ranking), while strains BS68, BS71 and BS73 demonstrated less biofilm development.

### Scanning electron microscopy (SEM) comparison of two *S. pneumoniae *isolates

**Figure 3:**
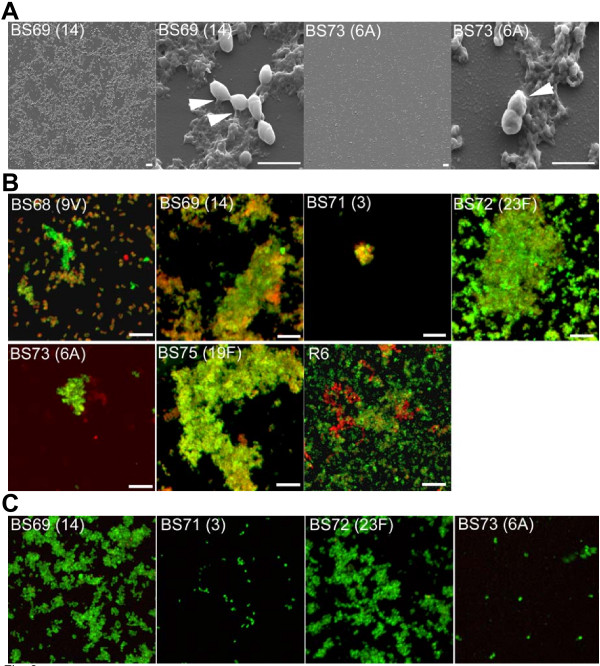
**Investigation of EPS matrix and antibiotic resistance in pneumococcal biofilms. 3A **Scanning electron microscopy of pneumococcal biofilms on polystyrene by BS69 (a high BFI strain) and BS73 (a low BFI strain) showing cluster morphology and evidence of extracellular matrix. Extracellular material can be seen on higher magnification in both clinical isolates, however more matrix material is visible with BS69 compared with BS73. Areas of extracellular material can be seen tethering *S. pneumoniae *cells to the surface (arrows). Scale bar = 10 μm and 2 μm. **Fig. 3B. **Strain variability in EPS distribution of pneumococcal biofilms by different clinical isolates demonstrated by lectin binding. Lectin (green fluorescence) and Syto 59 (red fluorescence) indicate binding of probes to carbohydrate or nucleic acid, respectively. Yellow indicates co-localization of the two probes. Images are maximum projections or reconstructed confocal stacks consisting of a series of x-y sections. Scale bar = 10 μm. **Fig. 3C. **Pneumococcal biofilms treated with azithromycin. High ranked BFI strains (BS69 and BS72) show large cell clusters still viable with the *Bac*Light LIVE/DEAD stain after 24 hours of antibiotic treatment. Low ranked BFI isolates (BS71 and BS73) on the other hand, show only a few viable attached cells (Scale bar = 8 μm.).

BS69 and BS73 were further examined to assess a high and a low ranked BFI isolate, using high resolution SEM. Figure 3A shows more widespread surface coverage by the high ranked BFI isolate, BS69 compared with BS73 and higher resolution images of the isolates showed BS69 cells surrounded by extensive extracellular material, anchoring pneumococcal cells to the surface. In contrast, SEM showed numerous punctate microcolonies attached over the surface in the low ranked BFI biofilm isolate, BS73. Thus, SEM results confirmed that these two isolates differed in the number of attached cells, but also in the extent of extracellular material attached to the surface.

### *S. pneumoniae *biofilms demonstrate a carbohydrate matrix

The multilayered *S. pneumoniae *biofilm towers of BS69, BS72 and BS75 observed with CLSM and SEM suggested that biofilm towers were held together by an extracellular matrix. Since fixation for SEM dehydrates the biofilm and collapses the matrix, we further tested pneumococcal biofilms for extracellular carbohydrate using a cocktail of 5 fluorescently-conjugated lectins and the nucleic acid probe, Syto59 under hydrated conditions. Figure 3B shows biofilms of each pneumococcal isolate grown for 8 days. High ranked BFI isolates (BS69, BS72 and BS75) exhibited a much greater degree of lectin binding, whereas low ranked BFI isolates BS71 and BS73 exhibited less lectin binding. BS68 showed more biofilm ultrastructure using the combination of lectins and Syto 59 than the other low ranked BFI strains, exhibiting some clusters stained with nucleic acid stain with large discrete patches bound with lectin, however the clusters were uniformly smaller than those of high ranked BFI strains (Fig. 3B). Biofilms of the unencapsulated strain, R6, also bound lectin. Colocalization of lectin and nucleic acid binding differed between strains when biofilms were evaluated with Imaris Colocalization software, using regression analysis (r^2 ^values). Averages of Pearson's coefficient showed greater colocalization of nucleic acid and carbohydrate specific probes with biofilms of BS69, BS72 and BS75 (Rr > 0.75) than BS68, BS71 and BS73 (Rr < 0.70), consistent with lectin binding correlating with higher order biofilm ultrastructure. High ranked BFI strains significantly differed from low ranked strains (P < 0.05).

### *S. pneumoniae *biofilms exhibit increased resistance to antibiotic treatment

To interrogate another fundamental feature of biofilm growth, pneumococcal isolates were tested for antibiotic resistance. Growth of planktonic pneumococci from each strain was inhibited (demonstrated by no turbidity in the culture wells compared to wells containing no antibiotic) by a concentration of 2 μg ml^-1 ^azithromycin. In contrast, 6 day biofilms of these strains required concentrations from 2 to 1000 fold higher to demonstrate growth inhibition by azithromycin (no turbidity) (Table 1). High ranked BFI isolates, BS69, BS72 and BS75, exhibited a 64, 1000 and 32-fold increase in the azithromycin concentration necessary to achieve no turbidity, indicating BFI correlated with increased resistance to antibiotic inhibition of bacterial outgrowth from biofilms. *In situ *assessment of 6 day biofilms treated with 20 μg ml^-1 ^of azithromycin for 24 hours further showed that all strains demonstrated viable cells after treatment, but high ranked BFI strains, BS69 and BS72, exhibited viable cells in large attached cellular clusters with few red cells (Fig. 3C). In contrast, azithromycin treatment of the low ranked BFI strains (BS71 and BS73) showed only a few viable attached cells.

**Table 1:** Concentration of antibiotic producing no turbidity after azithromycin treatment for *S. pneumoniae *clinical isolates grown in planktonic culture and as biofilms treated with a twofold serial dilution of azithromycin from 2 mg ml^-1 ^to 2 μg ml^-1 ^for 24 hours.

***S. pneumoniae *isolate**	**Serotype**	Planktonic IC* (μg ml^-1^)	**Biofilm IC^† ^(μg **ml^-1^)	**Fold Increase (-)**	**n**
BS 68	9V	< 2	4	> 2	6
BS 69^+^	14	< 2	125	> 64	8
BS 71	3	< 2	4	> 2	6
BS 72^+^	23F	< 2	2000	> 1000	6
BS 73	6A	< 2	4	> 2	6
BS 75^+^	19F	< 2	62	> 32	8

* Highest concentration of azithromycin producing no turbidity after overnight incubation with antibiotic with planktonic cells.

^† ^Highest concentration producing no turbidity after overnight incubation with antibiotic with biofilm cells.

Turbidity was scored by eye as well as by determining the highest concentration that was within the standard deviation of the optical density measurements of 6–8 replicate untreated controls (no antibiotic).

^+ ^Indicates high ranked BFI strain.

### *S. pneumoniae *clinical isolates have extracellular DNA in the matrix which is degraded with DNAse treatment

**Figure 4:**
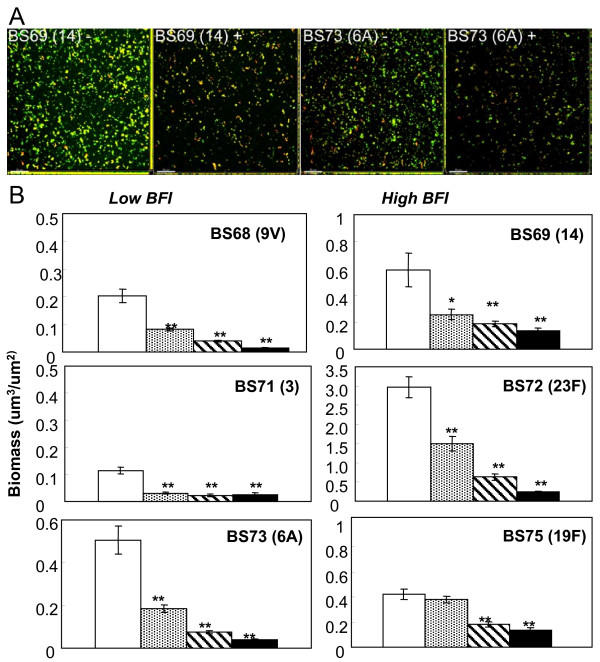
**DNA staining of the pneumococcal EPS matrix and disruption with DNase. Fig. 4A. **Biofilms stained with PicoGreen, a dsDNA stain, and Syto 59 shows that *S. pneumoniae *biofilms treated with 1000 μg ml^-1 ^Pulmozyme (+) were substantially reduced compared to untreated biofilms (-) in both high and low BFI strains. Scale bar = 30 μm. **Figure 4B**. Quantification of reduction in biofilm volume (total biomass) measured by COMSTAT upon treatment with different concentrations of Pulmozyme: Untreated controls □; 1 μg ml^-1 ^Pulmozyme ▩; 100 μg ml^-1 ^Pulmozyme ⊟; 1000 μg ml^-1 ^Pulmozyme ■. Bars show average of PicoGreen signal, which stains extracellular dsDNA, and Syto59, which stains intracellular nucleic acid. (Error bars represent standard error of the mean; n = 10; 5 randomly chosen microscopic fields from duplicate experiments. *Significantly different from untreated controls (P values < 0.05); ** (P values < 0.01). Statistical comparisons were made using one-way analysis of variance (ANOVA) (Excel 2000, Microsoft).

To further examine the composition of the pneumococcal biofilm matrix, biofilms were stained with PicoGreen and the nucleic acid dye, Syto 59. Figure 4A indicates that dsDNA is present extracellularly in the biofilm matrix for strains BS69 and BS73. To confirm the presence of extracellular DNA, when pneumococcal biofilms were treated with Pulmozyme^® ^(recombinant human DNase I) a significant loss of cells and biomass was observed (Fig. 4B). Biofilm degradation following Pulmozyme^® ^treatments was quantified using COMSTAT and results demonstrate that the biomass and average thickness of biofilms of all strains were significantly reduced by DNase treatment in a dose responsive manner (Fig. 4B). This was true for all strains regardless of their BFI, with the highest concentration of DNase resulting in reductions of over 90% in the average thickness of all strains except BS75 (Table 2). However, maximum thickness was less affected by DNase treatment in all of the strains, suggesting that the EPS matrix in biofilm towers consisted of non-DNA components.

**Table 2:** Degradation of *S. pneumoniae *Biofilms with DNase treatment.

**Strain**	**Pulmozyme concentration**				**[% Reduction^†^]**
	**0 (μg ml^-1^)**	**1 (μg ml^-1^)**	**100 (μg ml^-1^)**	**1000 (μg ml^-1^)**	
**Biomass (μm^3^/μm^2^)**					
***Low BFI***					
BS68 (9V)	0.20 (0.024)	0.08 (0.005)**	0.04 (0.003)**	0.01 (0.001)**	[95.0]
BS71 (3)	0.11(0.013)	0.03 (0.004)**	0.022 (0.005)**	0.02 (0.007)**	[81.8]
BS73 (6A)	0.51(0.065)	0.19 (0.019)**	0.08 (0.008)**	0.04 (0.003)**	[92.2]
***High BFI***					
BS69 (14)	0.59 (0.126)	0.26 (0.039)*	0.19 (0.020)**	0.14 (0.021)**	[76.3]
BS72 (23F)	2.97 (0.278)	1.50 (0.190)**	0.63 (0.083)**	0.24 (0.027)**	[92.0]
BS75 (19F)	0.42 (0.041)	0.38 (0.028)	0.18 (0.020) **	0.14 (0.017)**	[66.7]
**Average thickness (μm)**					
***Low BFI***					
BS68 (9V)	0.23 (0.032)	0.08 (0.006)**	0.03 (0.003)**	0.01 (0.002)**	[95.7]
BS71 (3)	0.19 (0.048)	0.03 (0.017)**	0.003 (0.000)**	0.004 (0.001)**	[94.7]
BS73 (6A)	0.46 (0.068)	0.16 (0.020)**	0.04 (0.005)**	0.02 (0.002)**	[95.7]
***High BFI***					
BS 69 (14)	0.46 (0.068)	0.16 (0.020)**	0.04 (0.005)**	0.02 (0.002)**	[91.3]
BS72 (23F)	5.23 (0.567)	2.23 (0.404)**	0.43 (0.073)**	0.15 (0.027)**	[97.1]
BS75 (19F)	0.39 (0.039)	0.26 (0.024)**	0.12 (0.017)**	0.058 (0.006)**	[85.1]
**Maximum thickness (μm)**					
***Low BFI***					
BS68 (9V)	3.6 (0.147)	3.23 (0.091)	2.44 (0.112)**	2.72 (0.275)**	[24.4]
BS71 (3)	5.2 (0.169)	3.72 (0.219)**	2.32 (0.099)**	2.00 (0.148)**	[61.5]
BS73 (6A)	5.28 (0.249)	3.2 (0.082)**	2.56 (0.110)**	2.56 (0.073)**	[51.5]
***High BFI***					
BS 69 (14)	7.12 (0.301)	4.72 (0.343)**	4.24 (0.193)**	3.6 (0.159)**	[49.4]
BS72 (23F)	10.08 (0.653)	4.96 (0.331)**	3.36 (0.160)**	3.68 (0.309)**	[63.5]
BS75 (19F)	4.20 (0.265)	3.84 (0.198)	3.04 (0.110)**	2.24 (0.110)**	[44.3]

Value = average (n = 10)

^†^Percent reduction of biofilm with highest dose of Pulmozyme.

(Standard error) * p < 0.05; ** p < 0.01

### *CpsA *expression is downregulated in pneumococcal biofilms

**Figure 5:**
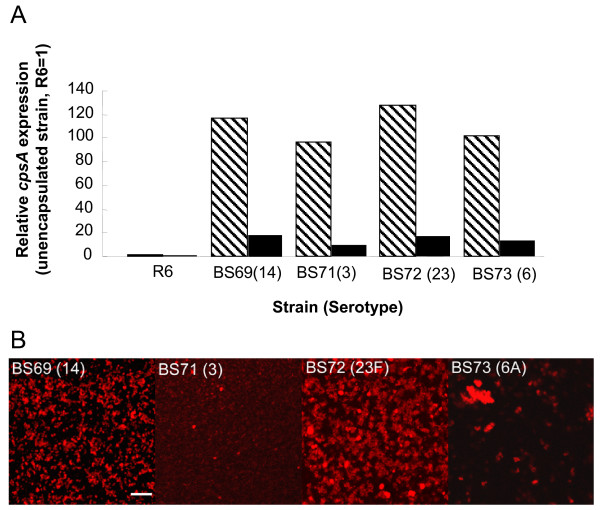
**Capsule expression during biofilm growth conditions in selected strains. Fig. 5A. ***cpsA*, the first gene in the pneumococcal capsule operon is expressed in each isolate over 100-fold relative to R6, an unencapsulated strain (hatched bars), but is downregulated when pneumococcal strains are grown as biofilms (solid bars). **Fig. 5B. **Immunostaining with anti-capsule specific antibody labeled with a secondary Texas red anti-rabbit antibody, shows that pneumococci express type specific capsule in the biofilm despite *cpsA *downregulation. Scale bar = 10 μm.

To investigate whether pneumococci in biofilms were encapsulated we examined *cpsA *expression under planktonic and biofilm growth conditions in 4 of the isolates (2 high ranked and 2 low ranked BFI strains) to see if capsule production was modulated during biofilm development. (All strains grown under planktonic conditions were confirmed to be positive for capsule by the Quellung (agglutination) reaction.) All planktonic grown encapsulated clinical isolates expressed more *cpsA *(~100 fold) than the unencapsulated R6 strain (Fig. 5A). However, expression of *cpsA *was downregulated in the biofilm relative to planktonic growth conditions in all strains, regardless of serotype or BFI. Expression of *cpsA *was higher overall in strains with a high BFI (BS69 and BS72) compared to low ranked strains (BS71 and BS73) with the relative fold reduction 6.3, 7.1, 10 and 7.7, respectively, indicating that BS71 exhibited the most extensive downregulation. *In situ *examination of biofilm-grown isolates using immunofluorescence with type-specific capsule (Fig. 5B) indicated that capsule-specific antibody binding was brightest in biofilm towers suggesting that pneumococci attached to the surface have a reduced amount of capsule. Taken together these results suggest that capsule expression undergoes complex modulation during biofilm growth.

## Discussion

The ability of upper respiratory pathogens including *S. pneumoniae *to persist in the nasopharynx and cause chronic disease upon the appropriate conditions may be associated with the ability to form biofilms on mucosal epithelium [7-9]. The presence of structurally complex bacterial biofilms is important because biofilms have been shown to exhibit increased resistance to host immune effectors and increased tolerance to antibiotic treatment [11,12,15], and therefore suggest that biofilms may contribute to the persistence of pathogens.

Previous reports have shown that *S. pneumoniae *can form biofilms *in vitro *using several different models of biofilm culture [16,20-26] including a study where CSLM data suggested that some of the strains used in the present study differed according to structural complexity [16]. Moreover in a pair of companion studies we have demonstrated that these strains have vastly different genomic complements [30] and produce significantly different disease phenotypes in an animal model of infection [31]. We therefore wished to further characterize biofilm formation of 6 of these pneumococcal strains by investigating the kinetics of biofilm formation, biofilm matrix composition, antibiotic resistance and capsule expression.

All clinical isolates developed biofilms containing viable, adherent cells over time under static conditions *in vitro*. However, biofilm development was highly variable among the different isolates. A multiparameter ranking, formulated to compare biofilm formation among the clinical strains using 3 standard assays commonly used to measure biofilm formation, identified two groups: those with a high biofilm forming index (BFI); BS69, BS72 and BS75, which attached quickly and produced more biofilm than the other strains, and those with a low BFI; BS68, BS71 and BS73 which produced biofilms consisting of adherent cells in small cell clusters with few towers. Our study suggests that a multi-assay approach for the quantification of biofilm formation better addresses strain to strain variation in the context of overall biofilm heterogeneity. Bacteria are frequently categorized as being positive or negative for biofilm formation typically using only a single assay such as crystal violet staining. However, our study suggests that such a binary distinction may lead to an oversimplified conclusion. Clearly, each of the six strains formed biofilms, but to different degrees. In the absence of clinical data correlating the degree of *in vitro *biofilm formation with infection severity and history, a biofilm etiology, even for a poor *in vitro *biofilm forming strain, should not necessarily be discounted. Not only was the BFI a useful construct to provide a clearer picture of the relative biofilm forming capacity of each strain, but it was also useful in testing putative biofilm characteristics, since a higher BFI correlated with greater binding with carbohydrate-specific lectins and antibiotic resistance *in vitro*.

Since SEM suggested that BFI was associated with biofilm structural complexity, biofilms from each pneumococcal strain were assessed for the presence of carbohydrate in the EPS matrix using lectin binding. High BFI strains (BS69, BS72 and BS75) exhibited the most lectin co-localization with the EPS matrix, while strains with a low BFI (BS71 and BS73) demonstrated reduced lectin co-localization, suggesting that polysaccharide is associated with larger multilayered aggregates of attached cells. BS68 exhibited some biofilm towers associated with nucleic acid and larger discrete patches of lectin binding (suggesting carbohydrate) than other low BFI strains. Lectin binding did not necessarily correlate with the pneumococcal capsule, since an unencapsulated strain (R6) which formed good biofilms, also bound lectin, suggesting that carbohydrate is associated with noncapsular EPS.

We further assessed another hallmark of biofilm development; antibiotic resistance. Six day pneumococcal biofilms with a low BFI exhibited a 2-fold increase in resistance to azithromycin compared to planktonic growth. However, strains with a high BFI required significantly higher concentrations to inhibit outgrowth from biofilms. *In situ *examination of 6 day biofilms treated with 20 μg ml^-1 ^of azithromycin (a concentration that was bactericidal for *S. pneumoniae *in epithelial cell culture) [32] showed large clusters of viable bacteria in high ranked BFI strains. Few viable attached cells were observed after azithromycin treatment in low ranked isolates. Although our study did not reflect a standardized minimum inhibitory concentration on biofilm bacteria due to the difficulty of assessing cell density within biofilms, these results support the hypothesis that biofilm-forming pneumococcal strains can persist in spite of antibiotic treatment and may contribute to chronic infection.

The presence of extracellular DNA (eDNA) in biofilms is now well documented in several types of bacterial biofilms including non-typeable *H. influenzae*, another major pathogen associated with chronic OM [33-36]. We therefore investigated if eDNA was present in the pneumococcal biofilm matrix. All strains showed evidence of eDNA shown by *in situ *staining of the matrix with the dsDNA stain, PicoGreen, and by a dose-dependent reduction of the pneumococcal biofilm biomass by recombinant human DNase I treatment using Pulmozyme^® ^(dornase alfa), used clinically to treat cystic fibrosis pneumonia. DNase-treated biofilms were significantly reduced in all pneumococcal strains when treated with the clinical dose of Pulmozyme^® ^(1 mg ml^-1^), exceeding 90% reduction in all but one strain. These results agree with those of Moscoso *et al*. who showed that DNase treatment reduced biofilms of the unencapsulated *S. pneumoniae *strain R6, and are consistent with the ability of *S. pneumoniae *to autolyse and release DNA [24,35].

Other *in vitro *studies with pneumococci have shown that capsule expression was associated with decreased biofilm formation [20,23,24,26] and some have reported that biofilm development may select for unencapsulated phenotypic variants [20,24,26]. However, *S. pneumoniae *is also known to phenotypically vary capsule production upon adherence to epithelial cells [27]. Therefore we hypothesized that the capsule operon might be downregulated in pneumococcal biofilms. Real time-qPCR results indicate that *cpsA *is downregulated in the biofilm up to 10-fold depending on the strain, compared to planktonic cultures regardless of the strain's BFI, suggesting that capsule production is variably modulated during sessile growth. *In situ *immunofluorescence staining with anti-capsule type-specific antibody further demonstrated that *S. pneumoniae *growing in biofilms was encapsulated and capsule immunostaining was brightest in biofilm towers compared to adherent cells. These results support the role of *cpsA *in capsule production [37,38]. Numerous reports demonstrate capsule phenotypic variation and show that variants with greater amounts of capsule colonize epithelial cells less efficiently [39-44]. Phenotypic variation of the capsule has been demonstrated microscopically during the initial stages of infection where adherent or invasive *S. pneumoniae *exhibited reduced amounts of capsule compared to cells not associated with epithelial cells [27]. Our results showing downregulation of *cpsA *in pneumococcal biofilms are consistent with observations that *S. pneumoniae *modulates capsule production upon adherence.

## Conclusion

Biofilm growth differed among six clinical isolates of *S. pneumoniae*. A multi-parametric assessment grouped isolates into two categories: those with a high biofilm forming index, which exhibited complex ultrastructure consisting of large multilayered biofilm towers; and those with a low BFI, which exhibited small compact microcolonies. BFI correlated with increased lectin binding and resistance to azithromycin. BFI did not correlate with the presence of DNA in biofilm EPS or modulation of capsule expression. DNase treatment resulted in a significant reduction of pneumococcal biofilms in all strains, however lectin binding to biofilm towers suggests the pneumococcal biofilm matrix is made up of both carbohydrate and DNA. Finally, *cpsA *was downregulated in all biofilm grown strains suggesting that capsule expression is phenotypically regulated upon biofilm development.

## Methods

### Isolation of strains and growth conditions

Clinical isolates of *S. pneumoniae *used in this study were obtained from nasal washes from symptomatic pediatric patients participating in a vaccine trial at Children's Hospital of Pittsburgh and isolated, cultured, serotyped and frozen as described previously [45]. All clinical isolates were encapsulated and were cultured identically. The six strains were: BS68 (serotype 9V); BS69 (serotype 14); BS71 (serotype 3); BS72 (serotype 23F); BS73 (serotype 6A); and BS75 (serotype 19F). Frozen stocks of *S. pneumoniae *were plated and individual colonies were picked and grown in THB to an optical density corresponding to 10^8 ^cells ml^-1^. Growth curves were obtained for each strain and OD and CFUs ml^-1 ^were correlated using regression analysis. Calibration curves for 1 × 10^8 ^CFUs ml^-1 ^and OD were used to estimate the inoculated number of *S. pneumoniae *cells and validated by plating as follows: BS68 (OD_600 _0.03); BS69 (OD_600 _0.04); BS71 (OD_600 _0.07); BS72 (OD_600 _0.03); BS73 (OD_600 _0.04) and BS75 (OD_600 _0.06). All experiments were carried out at 37°C, 5% CO_2_.

### Crystal violet assay for initial biofilm attachment

1 × 10^7 ^*S. pneumoniae *in THB were inoculated into 48-well plates (Corning, Lowell, MA), incubated for 24 hours and rinsed to remove non-adherent cells. One ml of 0.5% crystal violet (CV) solution (Sigma Aldrich) was added [31] and read at 600 nm using a Beckman DU 650 spectrophotometer (Beckman Coulter Inc., Fullerton, CA.). Background absorbance from medium blanks was subtracted from the values of triplicate samples from 5 independent experiments.

### Biofilm growth

1 × 10^8 ^ml^-1 ^*S. pneumoniae *was inoculated into MatTek culture plates (MatTek Corporation, Ashland, MA) or 6-well tissue culture plates (Falcon) and allowed to adhere overnight. *S. pneumoniae *biofilms were grown for 1, 3 and 6 days and culture medium was replaced daily with fresh, warm 1/5 strength THB. For colony forming unit (CFU) enumeration, *S. pneumoniae *biofilms were rinsed thrice with THB and adherent bacteria were detached using a cell scraper (Corning). One plate was harvested per time point and duplicate wells were combined (3 per 6 well plate), vortexed, diluted and plated in 2 independent experiments.

### Confocal Laser Scanning Microscopy

Pneumococcal biofilms were also visualized directly using CLSM and the *Bac*Light Bacterial Viability Kit. Biofilms were grown for 1, 3, and 6 days and stained according to the manufacturer's directions, rinsed, immersed in Hank's balanced salt solution (HBSS) with Ca^+2 ^and Mg^+2 ^and immediately examined with a Leica DM RXE microscope attached to a TCS SP2 AOBS confocal system (Leica Microsystems, Exton, PA) using a 63× water immersion lens and the sequential scanning mode. Images were analyzed using the Leica LCS software, COMSTAT [46] and Imaris Software (Bitplane; St. Paul, MN).

### Scanning electron microscopy (SEM)

Biofilms were processed for SEM by dehydration in a graded ethanol series. Samples were sputter coated with 200 angstroms of gold using a Hummer VII Sputtering System (Anatech Ltd., Alexandria, VA), visualized at an accelerator voltage of 5 kV using a Tescan Mira field emission scanning electron microscope (Tescan USA, Cranberry Township, PA) and digitized TIFF images were collected.

### EPS Carbohydrate binding with fluorescent lectin probes

To assess the extent and distribution of carbohydrate in the pneumococcal biofilm matrix, cultures were grown for 8 days as above, rinsed and stained with a cocktail of 5 lectins consisting of Alexa 488-conjugated lectins (Invitrogen) at the following concentrations: 50 μg ml^-1 ^of *Canavalia ensiformis *(Con A) specific for α-mannopyranosyl and α-glucopyranosyl residues, 125 μg ml^-1 ^of (Wheat germ agglutinin) WGA specific for N-acetyl glucosamine and N-acetylneuraminic acid, 100 μg ml^-1 ^of *Griffonia simplicifolia *(GS II), specific for terminal α- and β- linked *N*-acetyl-D-glucosaminyl residues; 125 μg ml^-1 ^of (Soybean agglutinin) SBA, specific for terminal β-galactose; and 75 μg ml^-1 ^of (Peanut agglutinin) PNA, specific for terminal α- and β-*N*-acetyl-galactosamine and galactopyranosyl residues in HBSS. Three μl of Syto 59 nucleic acid stain was added per ml of the cocktail. Cultures were stained for 35 minutes rinsed to remove unbound lectin and nucleic acid probe and sequentially scanned using the 488 and 633 nm laser lines to minimize channel cross-talk for co-localization analysis.

### COMSTAT image analysis

Quantitative analyses of the CLSM images by the COMSTAT computer software was performed on day 6 biofilms stained with *Bac*Light [46], or with PicoGreen, a dsDNA stain [33] as described. The biofilm parameters, biomass (biovolume), maximum and average thickness, and roughness coefficient (an indicator of biofilm heterogeneity) were assessed using a minimum of 5 different images per plate from 2 independent experiments for each isolate.

### Statistical analyis

Statistical comparisons were made using one-way analysis of variance (ANOVA) (Excel 2000, Microsoft). Differences were reported statistically significant for P < 0.05.

### Calculation of a "biofilm forming index" (BFI)

To combine the information from the 3 biofilm assays (crystal violet, 6 day CFU/cm^2 ^count and COMSTAT data) to give an overall assessment of the degree of biofilm formation of each pneumococcal strain, we used a simple normalized ranking system based on a "biofilm forming index" (BFI) for each assay. The Shapiro-Wilk normality test (The R Project for Statistical Computing: R statistical software version 2.6.2 (2008 02 08) freely available at ) was used to determine whether the data sets needed to be transformed to achieve a normal distribution (P > 0.05). Of the data sets for the 6 pneumococcal strains, 4 of 6 were normal for the CV assay and viable cell count, and 1 of 6 was normal for COMSTAT. Log_10 _transformation of the data resulted in all data sets conforming to a normal distribution (P > 0.05). The BFI for each of the individual assays for each of the six strains was determined using:

(1) BFI_OD _or BFI_CFU _or BFI_COMSTAT _= (Log_(10) _Strain value - Log_(10) _lowest value)/(Log_(10) _highest value - Log_(10) _lowest value)

Thus each strain was assigned a value ranging from "I", for the least biofilm, to "1" for the most biofilm.

The data from the 3 assays was combined to find the overall combined (BFI) from:

(2) BFI = (BFI_OD _+ BFI_CFU _+ BFI_COMSTAT_)/3

### Antibiotic treatment of *S. pneumoniae *planktonic cells and biofilms

Planktonic cultures were grown to mid-log phase and incubated with twofold dilutions of azithromycin ranging from a concentration of 2 mg ml^-1 ^to 2 μg ml^-1 ^in 1/5 THB for 24 hours in 24-well polystyrene plates (BD, Franklin Lakes, NJ). Biofilms were grown in 24-well plates for 6 days whereby non-adherent cells were removed to a new 24 well plate and incubated with twofold dilutions of azithromycin to test if nonattached cells at this period of culture were resistant to azithromycin. Spectrophotometric readings were taken at 595 nm using a GENios plate reader and also scored visually. The concentration of azithromycin that showed a lack of turbidity corresponding to the spectrophotometric reading of the negative control (THB only) was interpreted as indicating the minimum concentration of antibiotic that inhibited bacterial growth. Biofilms were exposed to identical concentrations of azithromycin and incubated as for planktonic cells; rinsed with THB and incubated for a further 24 hours. Visual and spectrophotometric readings of duplicate wells were taken in 3 different experiments and the lowest concentration that indicated no turbidity (inhibition of planktonic growth from the biofilm or 'showering') was interpreted as the minimum concentration of antibiotic that inhibited bacterial outgrowth from the biofilm. Additionally, *S. pneumoniae *biofilms were grown in MatTek plates for 6 days, rinsed and treated with 20 μg ml^-1 ^of azithromycin for 24 h [32], stained with *Bac*Light kit and imaged *in situ*.

### Pulmozyme^® ^(DNase 1) experiments

Six day biofilms for each strain were incubated for 15 minutes at RT in the dark with Pulmozyme^® ^(dornase alfa), a synthetic DNase I (supplied as 1 mg ml^-1 ^solution), (Genentech Inc, San Franscisco, CA) in duplicate at each of three concentrations (1 μg ml^-1^, 100 μg ml^-1 ^and 1 mg ml^-1^). Untreated controls were incubated with 200 μl of buffer alone (8.7 mg ml^-1 ^NaCl-0.15 mg ml^-1^CaCl_2_). Plates were rinsed and stained for 15 minutes with 100 μl each of PicoGreen (1 μl ml^-1^) and Syto 59 (3 μl ml^-1^) (Invitrogen) [36] in HBSS, rinsed and imaged.

### Isolation of RNA

To assess capsular gene expression in clinical isolates, real-time quantitative PCR (RT-qPCR) was used to measure the transcripts of the pneumococcal capsular gene, *cpsA *[29]. For the isolation of RNA, BS69, BS71, BS72 and BS73 and the unencapsulated strain R6, were grown to late log phase (planktonic) or harvested from 6-well plates after 6 days (biofilm). Total cellular RNA was then isolated as previously described [47]. RNA preparations were tested for contaminating DNA by no-reverse-transcriptase PCR reactions.

### Real-time quantitative PCR

Oligonucleotide primers and TaqMan probes (Fam-labeled TaqMan TAMRA probes) used for RT-qPCR were designed with Primer Express 2.0 software (ABI Prism; PE Biosystems, Framingham, MA.) to amplify gene fragments with an optimal size of 60–130 bp. Sequences of primer sets and corresponding probes used are listed in Table 3. Measurements of relative levels of gene expression were done by RT-qPCR as described [47].

**Table 3:** Oligonucleotide primers and TaqMan probes used in RT-qPCR analysis of gene expression

**Gene**	**Sequences (5'→3')**		**Source**
	**Primer**	**Taqman Probe (Fam-Tamra)**	
CpsA	F: CTTTGCAGTACAGCAGTTTGTTG	ACTGACCAATCGTTTAAATG	Ref. [25]
	R: TCTGCTAAAACAGCGACACTGA		
gyrB*	F: CCAAACCGACTATTCAGCGTTAT	TGAAATGGACGATCATCAGCTGTGGGA	In this study
	R: TGTTCGGGATCCATGGTTGT		

*gyrB was used as endogenous control.

PCR reactions were done in 15 μl reaction volumes containing the reaction mixtures as follows: 1.5 μl cDNA; 7.5 μl universal PCR master mix; 1.2 μl forward primer (800 pM); 1.2 μl reverse primer (800 pM); 1.2 μl Taqman probe (160 pM) and 2.4 μl nuclease-free de-ionized H_2_O. PCR amplification parameters were as follows: 50°C for 2 minutes, followed by 95°C for 10 minutes, and then 45 cycles of 95°C for 15 s and 60°C for 1 minute. All qPCR reactions were done in triplicate and the mean C_T _was used for analysis of results. The constitutively expressed gene for DNA gyrase (*gyr*) was used as an endogenous control as described previously [25]. The ΔC_T _values were determined by subtracting the *gyr *C_T _value from the gene-specific C_T _values. The ΔΔC_T _value was calculated by subtracting the ΔC_T _value obtained with the ΔC_T _calibrator value. To verify the absence of contaminating DNA, each RT-qPCR experiment included controls that lacked template cDNA or reverse transcriptase. The amount of target gene transcript was expressed as the difference (n-fold) from the amount of the control gene (2^-ΔCT^), where ΔC_T _represents the difference in threshold cycle between the target and control genes. Analysis of expression of each gene was done based on at least 2 independent experiments. Twofold or higher changes in gene expression were considered significant.

### Capsule determination

Planktonic cultures of BS68, BS71, BS72 and BS73 were incubated with capsule-specific antiserum according to supplier instructions (Statens Seruminstitut, Copenhagen, Denmark). For qualitative determination of pneumococcal capsule, biofilms were rinsed, scraped and incubated with type-specific capsule antibodies as for planktonic cells. Additionally, 8 day biofilm cultures were rinsed and examined *in situ *using CLSM after incubation with polyclonal rabbit anti-capsular antibody and a secondary AffiniPure F(ab)_2 _fragment Texas Red-conjugated donkey antirabbit IgG (H+L chain) antibody (Jackson ImmunoResearch, West Grove, PA).

## Authors' contributions

LHS designed and developed the pneumococcal biofilm assays including the lectin binding assays and characterization of biofilm matrix. The biofilm experiments were carried out by LN and CJ. JCP, GDE and LHS designed the pulmozyme experiments and BD carried out the pulmozyme experiments. RT-qPCR experiments were designed by LHS and developed and carried out by KS. COMSTAT analysis was done by BD and DN. Statistical analysis was done by PS, who additionally developed the biofilm metric. LHS wrote the manuscript and GDE and PS provided discussion, editing and critical reading of the manuscript. All authors read and approved the manuscript.
